# A deep learning framework for classifying microglia activation state using morphology and intrinsic fluorescence lifetime data

**DOI:** 10.3389/fninf.2022.1040008

**Published:** 2022-12-16

**Authors:** Lopamudra Mukherjee, Md Abdul Kader Sagar, Jonathan N. Ouellette, Jyoti J. Watters, Kevin W. Eliceiri

**Affiliations:** ^1^Department of Computer Science, University of Wisconsin, Whitewater, WI, United States; ^2^Laboratory for Optical and Computational Instrumentation, Department of Biomedical Engineering, University of Wisconsin Madison, Madison, WI, United States; ^3^Department of Comparative Biosciences, University of Wisconsin Madison, Madison, WI, United States; ^4^Morgridge Institute for Research, Madison, WI, United States; ^5^Department of Medical Physics, University of Wisconsin Madison, Madison, WI, United States

**Keywords:** microglia activation state, deep learning, fluorescence lifetime, morphology, LSTM

## Abstract

Microglia are the immune cell in the central nervous system (CNS) and exist in a surveillant state characterized by a ramified form in the healthy brain. In response to brain injury or disease including neurodegenerative diseases, they become activated and change their morphology. Due to known correlation between this activation and neuroinflammation, there is great interest in improved approaches for studying microglial activation in the context of CNS disease mechanisms. One classic approach has utilized Microglia's morphology as one of the key indicators of its activation and correlated with its functional state. More recently microglial activation has been shown to have intrinsic NADH metabolic signatures that are detectable *via* fluorescence lifetime imaging (FLIM). Despite the promise of morphology and metabolism as key fingerprints of microglial function, they has not been analyzed together due to lack of an appropriate computational framework. Here we present a deep neural network to study the effect of both morphology and FLIM metabolic signatures toward identifying its activation status. Our model is tested on 1, 000+ cells (ground truth generated using LPS treatment) and provides a state-of-the-art framework to identify microglial activation and its role in neurodegenerative diseases.

## 1. Introduction

Microglia are Central Nervous System (CNS) resident macrophages that play important roles in many neuropathologies (Watters et al., 2005; Garden and Möller, 2006; Tambuyzer et al., 2009; Charles et al., 2011). They are involved in brain development, response to injury and infection as well as maintenance of the healthy neural microenvironment. Due to their central role in so many CNS processes and neurodegenerative diseases, it is important to understand the function of microglia in a number of scenarios including microglia activation. In this respect, accurate quantitative imaging and computational tools are needed to identify morphological signatures specific to microglia and understand how they correlate with their activation status.

**Figure 1:**
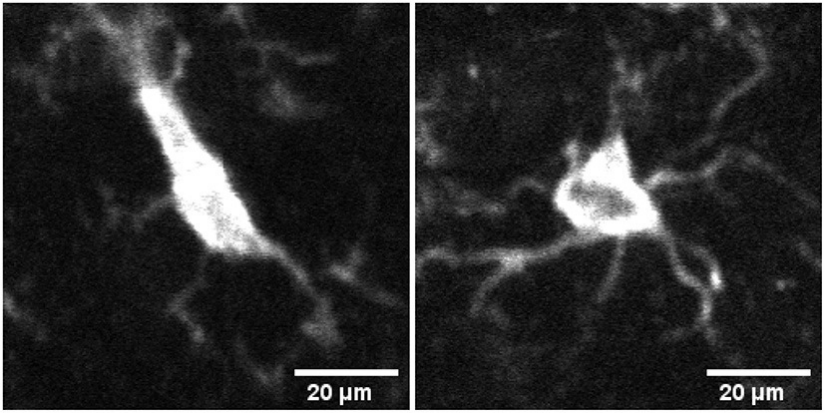
Image of microglia in activated **(left)** and surveillant/resting **(right)** state from mid brain section of mouse brain tissue samples.

Microglia react quickly to changes in their environment by exhibiting morphological changes. For example, the change in microglia's activation status is reflected in its gradual morphological transformation from highly ramified into less ramified often amoeboid state (see Figure 1). Table 1 summarizes the recent literature of morphological classification of microglia. These morphological changes are often closely related to their functional states, and for this reason, microglial morphology is often utilized to infer their activation status, and to study their involvement in virtually all brain diseases (Heindl et al., 2018). Until recently, available microscopic methods have been unable to capture the extent of these changes in an automated manner, relying mostly on manual assessments which can be prone to error. In the last few years, studies such as (Zanier et al., 2015; Leyh et al., 2021) have performed microglia morphology classification using machine learning methods by using carefully chosen shape features. However, because the commonly used feature set is typically limited to a few, manually chosen shape parameters, there may be selection bias which can compromise the resulting classifications.

**Table 1:** Table summarizing a few methods related to classification of microglia phenotypes from literature.

**Torres-Platas et al. (2014)**	**Identified four main phenotypes based on morphology: ramified, primed, reactive and amoeboid in humans**
Reemst et al. (2022)	Identified two morphological subtypes in PBS-injected mice, a small cell soma and long branched ramifications or a larger cell soma and thicker, branched ramifications
Leyh et al. (2021)	Identified four microglial morphologies, ramified, rod-like, activated and amoeboid microglia using a mouse model of ischemic stroke using machine learning
Verdonk et al. (2016)	Using the knock-in mouse model, found that in resting state, microglial cells were distributed in four microglial sub-populations with a regional pattern and specific behavior.

Additional review can be found in Schwabenland et al. (2021).

Furthermore, given the important role of microglia plays in all neural diseases, accurate tools for detecting their function beyond morphological alterations are also necessary. In this respect, it has been shown by Sagar et al. (2020b) that microglia metabolic state have unique metabolic fluxes which can be detected by changes in reduced nicotinamide adenine dinucleotide (NADH) *via* fluorescence lifetime imaging microscopy (FLIM) (Lakowicz et al., 1992a, 1992b). See Mechawar et al. (2022) for a survey of proinflammatory, anti-inflammatory and metabolic pathways in microglia as well as Rahimian et al. (2021) to understand the diversity of microglial phenotype and function in psychiatric diseases. FLIM can probe the cellular microenvironment of the fluorescent NADH in a label-free manner which does not change the metabolic signature. Furthermore, recent machine learning methods have shown that FLIM based lifetime data capturing the metabolic alterations, can be used to both differentiate microglia from other CNS cell-types using deep learning approaches (Sagar et al., 2020a; Mukherjee et al., 2021) as well as identify their activation status (Sagar et al., 2020b). These studies show promise in the use of computational tools for analyzing FLIM data to understand the functional role of microglia in the CNS.

**Figure 2:**
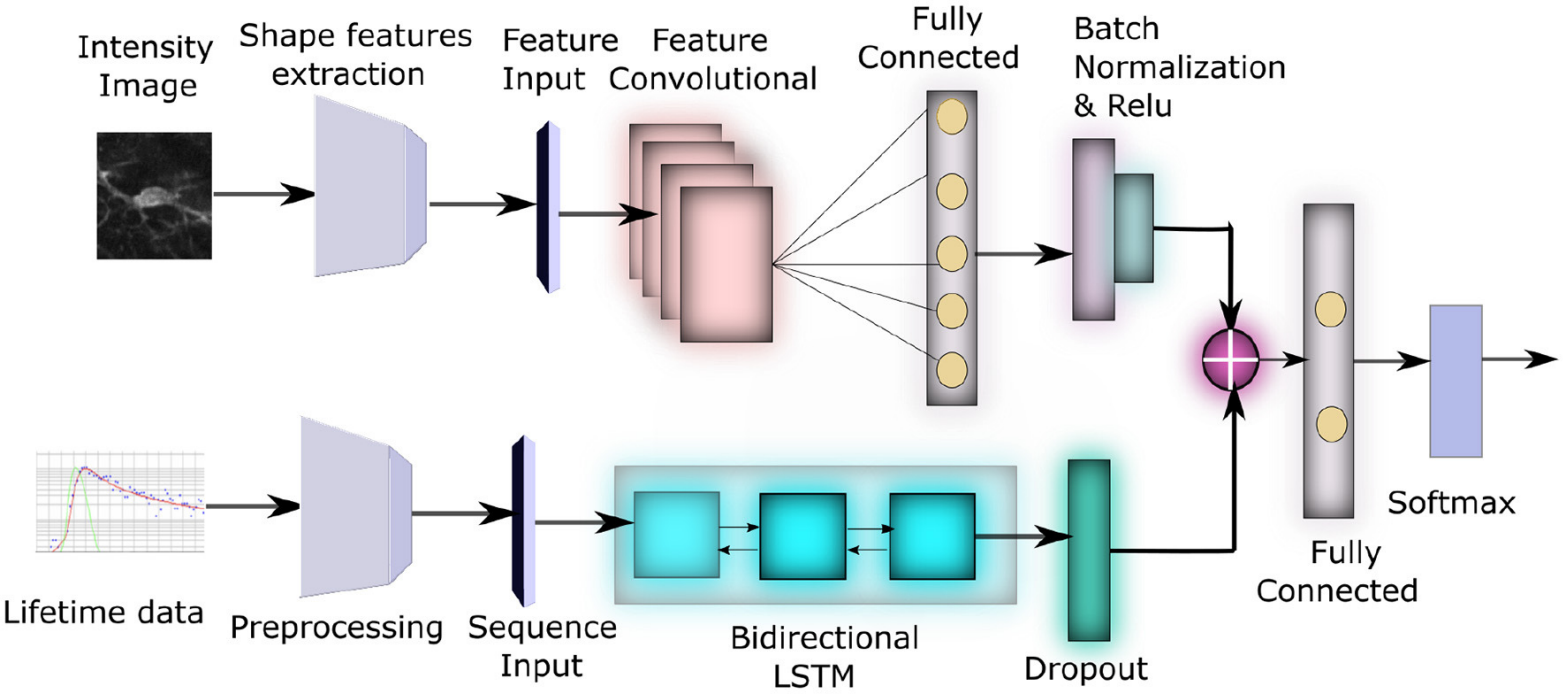
Block diagram of our network architecture: The top subnetwork inputs the intensity image from each sample and converts it to a feature vector using a Feature Convolutional Network. The bottom subnetwork input the lifetime data for each sample, converts it to 3D time-series tensor of average lifetimes across all pixels in the preprocessing step, which is then passed through a bidirectional LSTM. These two feature sets are eventually concatenated to produce the final classification.

It has been recently shown that different microglia functional phenotypes are associated with both distinct metabolic pathways as well as specific morphological changes. Voloboueva et al. (2013) and Orihuela et al. (2016). However a complete computational study investigating both the role of morphology as well as metabolism toward identifying activation state of microglia has not been performed until now. In this paper, we propose an unified deep neural network method to study the effect of both morphology as well as lifetime toward identifying the activation status of microglia. The framework of the model is shown in Figure 2. First, we extract a number of shape features from segmented images of microglia to characterize their morphological properties. This along with the lifetime data from the same images is inputted to a neural network, consisting of Long Short Term Memory (LSTM) (Yu et al., 2019) and convolutional sub-networks, which process each type of data, which are subsequently combined to yield a joint classification framework that can distinguish between activated and resting microglial cells. Experimental evaluations show that this leads to a highly accurate network compared to morphology or lifetime considered standalone and can classify the activation state correctly across a number of samples. This combined morphology-FLIM metabolism deep learning framework provides a computationally efficient approach to identify the activation state of microglia automatically, which can be useful to analyze its role in neurodegenerative diseases.

## 2. Methods

The proposed model combines CNN for learning features from the morphology data with LSTM for learning temporal dependencies in the lifetime data, to derive the classification of activated microglia from resting. We describe the details of each approach next.

### 2.1. Morphological features and network

The morphology of microglia is one of their more outstanding characteristics. Microglia remain in a *resting or surveillant state* in the normal brain, but upon the detection of any brain lesion or injury, they obtain an “activated” state which displays more inflammatory features. This change in state also manifests in a change of morphological characteristics- from a more ramified structure in resting state to a more ameboid shape in the activated state. Quantifying such changes can be the key to identifying the activation state of microglia. Here, we aim to capture the changes in morphological characteristics of microglia, by obtaining a number of shape features, which are then concatenated and passed as input to a convolutional neural network (CNN). The CNN transforms the input features to a different feature space, which is more conducive for learning the classes. To obtain the input shape features, we segmented the microglia from background using the WEKA segmentation toolbox in Fiji (Arganda-Carreras et al., 2017). We used a diverse set of features from moment based features to contour and transform based features as well as shape signature based 1D features. We describe these features next.

#### 2.1.1. Zernike invariant moment features

The Zernike moment (Khotanzad and Hong, 1990; Hwang and Kim, 2006) is a type of the orthogonal invariant (to translation, rotation and scale) moment on the unit sphere and is the most commonly used in image shape feature extraction and description. These are designed to capture both global and geometric information about objects of interest in the image. To compute the Zernike moments of an image, the range of the image is first mapped to the unit sphere with its origin at the image's center. The pixels falling outside the unit sphere are not used in the computation process. Let *f* (*r*, θ) be the two dimensional image intensity function using polar coordinates, then *Z*_*mn*_, the Zernike moment of order *m* and repetition *n* is denoted by


(1) $$\begin{array}{l} Z_{mn}=\frac{m}{\pi }\underset{r}{\sum }\underset{\theta }{\sum }f\left(r,\theta \right)V^{*}\left(r,\theta \right) \end{array}$$

Where $V_{mn}^{*}\left(r,\theta \right)$ is the complex conjugate of Zernike polynomial *V*_*mn*_(*r*, θ), defined as follows Liu et al. (2007):


(2) $$\begin{array}{l} V_{mn}\left(r,\theta \right)=R_{mn}\left(\theta \right)\exp\left(\sqrt{-1}r\theta \right) \end{array}$$

Where *m* and *n* are nonnegative integers with *n* ≥ *m* ≥ 0 and the orthogonal radial polynomial *R*_*mn*_(θ) is given as


(3) $$\begin{array}{l} R_{nm}(\theta )=\overset{\frac{n-m}{2}}{\underset{k=0}{\sum \;}}\frac{{\left(-1\right)}^{k}(n-k)!}{k!\left(\frac{n+m}{2}-k\right)!\left(\frac{n-m}{2}-k\right)!}\theta^{n-2k} \end{array}$$

Note that the different order of the Zernike moments can be computed *via* (Equation 1) by varying the order or keeping the order fixed and varying the repetition. It has been shown that the lower-order Zernike moments are useful to represent the whole shape of the image whereas the high-order Zernike moments can describe the details. In our approach, we compute the top 10 Zernike moments.

#### 2.1.2. Chord length histogram

**Figure 3:**
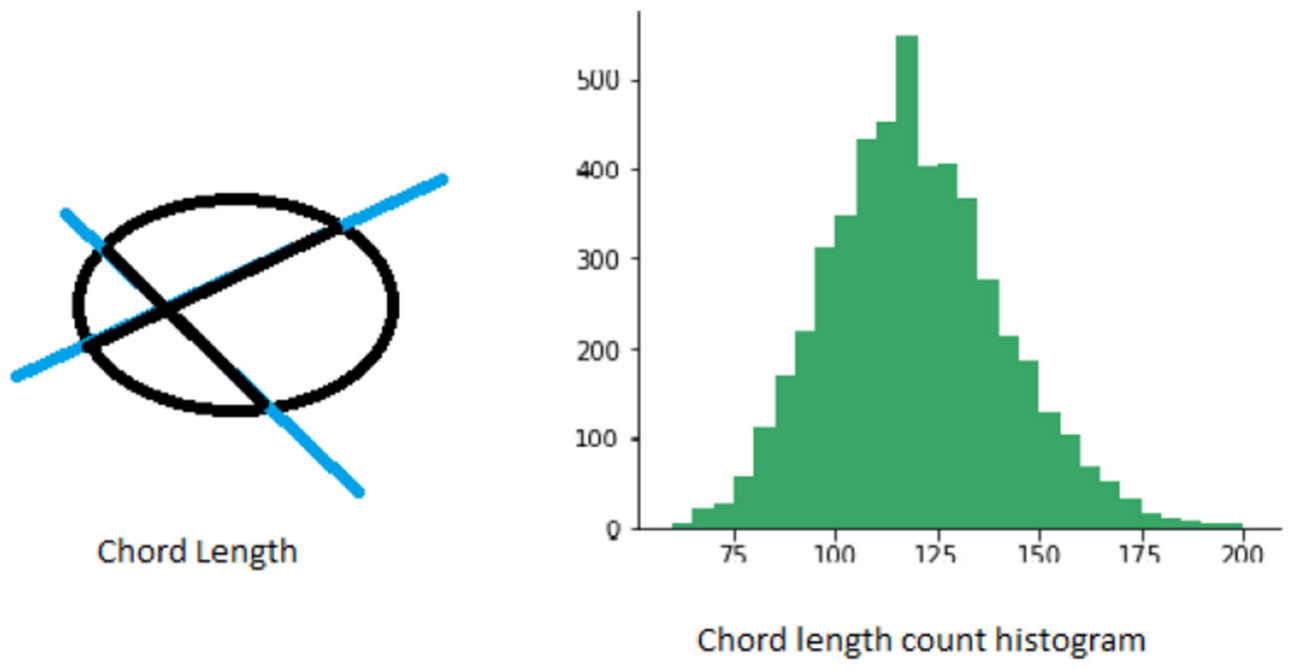
Figure illustrating chord length histograms.

Chord length histogram analysis (Agimelen et al., 2016) corresponds to finding the distribution of all chord lengths in different directions in a given shape. The chords are defined by the parts of lines within the contour of a binary shape. For each boundary point *p*, its chord length function is the shortest distance between *p* and another boundary point $\hat{p}$ such that line $p\hat{p}$ is perpendicular to the tangent vector at *p*. The chord length function is invariant to translation and its centroid is not biased by boundary noise. A shape can be represented by a discrete set of chords sampled from its contour. The number and length of chords obtained in different directions is generally not the same. Therefore, one way to capture this information more effectively is to calculate the distribution of chord lengths in the same direction in a spatial histogram. See Figure 3 for an illustration.

#### 2.1.3. Elliptical fourier features

This method is based on computing Fourier coefficients to describe a closed contour by a function. First the boundary is extracted from the segmented image of the object. Then the contour edge is encoded using Freeman encoding of closed contour, which yields a chain code, where each integer represents an oriented vector in a specific direction. The length of each of these vectors and their projections on the *x* and the *y* axes are computed, which is then used in the calculation of the elliptic harmonics. Elliptical Fourier analysis then approximate a closed contour as a sum of elliptic harmonics. Let *x*(*t*) be the *x* projection of the complete contour. Then,


$$\begin{array}{l} x\left(t\right)=x_{0}+\overset{N}{\underset{n=1}{\sum }}x_{n}\left(t\right)\\ x_{0}=\frac{1}{T}\int_{0}^{T}x\left(t\right)dt\\ x_{n}\left(t\right)=\alpha_{n}\cos\left(\frac{2n\pi t}{T}\right)+\beta_{n}\sin\left(\frac{2n\pi t}{T}\right)\\ \alpha_{n}=\frac{2}{T}\int_{0}^{T}x\left(t\right)\cos\left(\frac{2n\pi t}{T}\right)dt\\ \beta_{n}=\frac{2}{T}\int_{0}^{T}x\left(t\right)\sin\left(\frac{2n\pi t}{T}\right)dt \end{array}$$

Here *T* is the period of *x*(*t*). We followed the strategy of Kuhl and Giardina (1982) who used four Fourier coefficients for each of *N* harmonics. Then an inverse process is employed to identify the closed contour as *k* elements, which can be found as a function of the *N* harmonics. In addition, phase shift is employed so that the representation does not depend on starting point. See Kuhl and Giardina (1982) and Ballaro et al. (2002) for details regarding specific computations.

#### 2.1.4. General shape features

In addition to the above, we also computed a number of 1D general shape features, which describe the high-level geometric properties of the objects. These include area, perimeter, eccentricity, major and minor axis length, bounding box and equivalent diameter. Such features can be used as filters to eliminate false positives and are generally combined with other shape descriptors (as in our case) to discriminate shapes.

#### 2.1.5. Network for learning shape features

**Figure 4:**
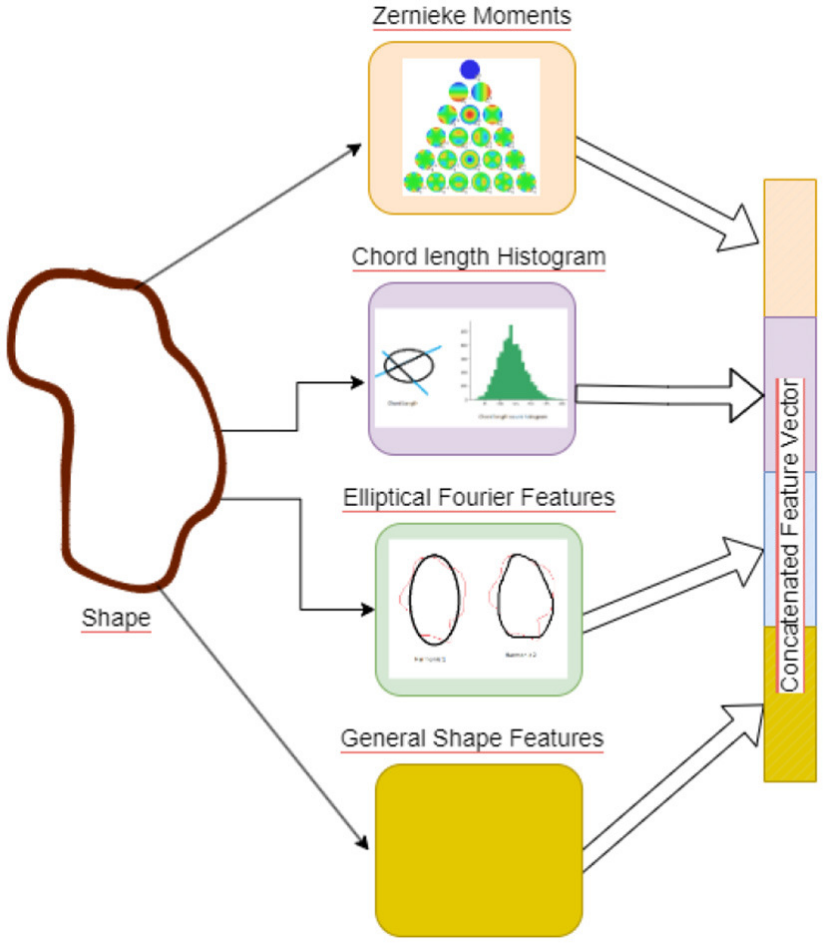
Figure illustrating the Shape Features extraction pipeline. Illustrative picture of Zernike Moments obtained from https://github.com/sgabriela/Zernike-Moments.


Shape features are concatenated to generate a 67 dimensional feature vector, see Figure 4, the features are then mean centered and passed through a feature convolutional neural network. The first layer in this network is a feature input layer. This is followed by a Feature Convolution Network as described by Hu (2021) which extends the idea of convolution to tabular feature data. Normally the convolution process is applied to the spatial region of an image using a kernel. To extend this to tabular features, we use the method proposed by Hu (2021) for combining pairs of features to create new features.

(4) $$\begin{array}{l} \phi_{ij}\left(x\right)=\alpha_{i}x_{i}+\beta_{j}x_{j} \end{array}$$

Where *x*_*i*_ (and *x*_*j*_) are the *i*th (and *j*th) dimensional feature and α_*i*_ and β_*j*_ are the weights of the kernel. There is one kernel parameter (α and β) for each feature in the convolution operation. Therefore, if the number of features are *n*, this produces a feature map of size *C*(*n*, 2). The network outputs ϕ as the output of feature convolution layer, which is then added with *x*, the original features, making it a function of both original and convolved features. In our network, this is followed by a Fully Connected (FC) layer (with 50 hidden units), a batch normalization layer, and Rectified Linear activation (ReLU) layer. The output of the ReLU layer is concatenated with the output of the LSTM to form a joint network.

### 2.2. Lifetime data and LSTM subnetwork

Fluorescence Lifetime image microscopy (FLIM) (Lakowicz et al., 1992a, 1992b) is an imaging technique which is used to visualize physiological properties in living cells by measuring the time a molecule (excited by a photon) remains in its excited state on an average before returning to its ground state and emitting a photon. This lifetime of the molecule is then calculated using an exponential decay function and is used to localize specific fluorophores. Also, FLIM can use intrinsic fluorescence of NADH to probe cellular micro-environment in a label-free manner which does not alter the cellular signature. FLIM related monitoring of microglial metabolic alterations can be used to both differentiate microglia from other CNS cell-types (Sagar et al., 2020a; Mukherjee et al., 2021) as well as identify their functional state (Sagar et al., 2020b).

We first describe the mathematical representation of the fluorescent decay curve obtained by FLIM, followed the architecture of the LSTM subnetwork used to process this data. Assume a single image is under consideration of size *m* × *m*. The measured fluorescence intensity decay data (*Y*_*t*_) for a given lifetime component *t* is given by the convolution of the tissue fluorescence response signal (*I*_*t*_) with the excitation light pulse [part of the instrument response (*F*_*t*_)] along with some additive noise (ϵ_*t*_). This relationship can be written as


(5) $$\begin{array}{l} Y_{t}=I_{t}⊗F_{t}+\epsilon_{t} \end{array}$$

Where ⊗ represents convolution of the two signals and *Y*_*t*_ (as well as *I*_*t*_ and ϵ_*t*_) are of size *m* × *m*. The function *I*_*t*_ can be approximated as a multi-exponential decay function, with multiple components. Here we use bi-exponential decay function to represent the signal, because in practice, it is enough to closely approximate the signal and can be written as


(6) $$\begin{array}{l} I_{t}\sim a_{1}\exp\left(\frac{-t}{\tau_{1}}\right)+a_{2}\exp\left(\frac{-t}{\tau_{2}}\right) \end{array}$$

Where *a*_1_, *a*_2_ and τ_1_, τ_2_ represent the amplitude and the lifetimes for each exponential sub-function in this bi-exponential function. We now extend this data to multiple images by using the superscript *i* to denote the image number. The lifetime data ${\left\{Y_{t}\right\}}^{i}$ (without the noise) is collected for the same lifetime component *t* ∈ *t*_1_, …*t*_*p*_ for all images *i* ∈ 1…*n* in our dataset. ${\left\{Y_{t}\right\}}^{i}$ is then vectorized and binned into *h* bins to generate a vector ${\left\{X_{t}\right\}}^{i}$ in *R*^*h*^, which forms the input to the following Long Short Term Memory or LSTM network. The same number of bins are used across all images, making it feasible to compare them as multiple time-series data. The target class label *y* is of size *n*. An intuitive way to understand this data is that there are *h* dimensional features for each image which evolve over lifetime components and can be used to understand group differences between the two classes under consideration.

**Figure 5:**
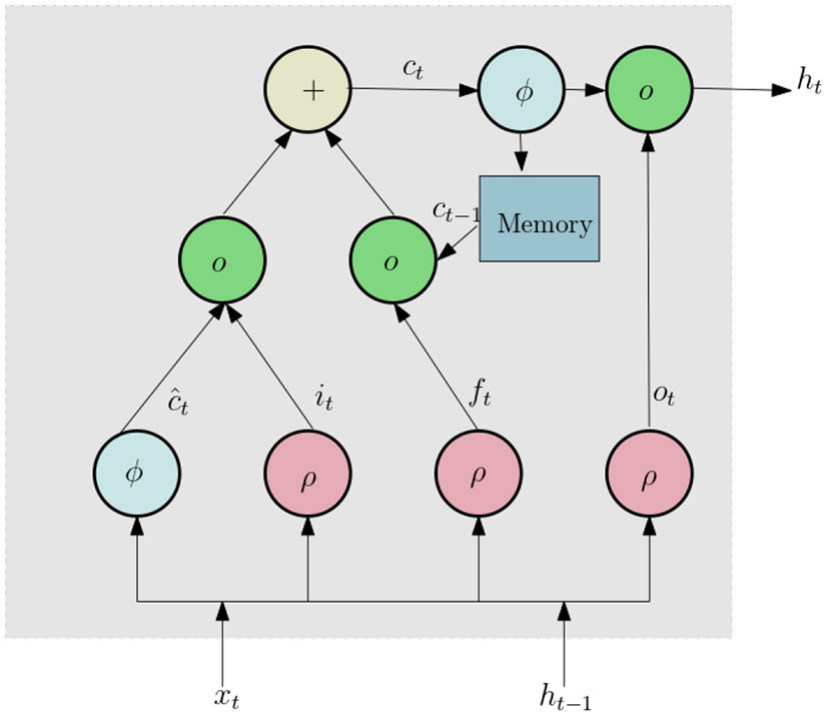
Schematic diagram of LSTM unit.

We now discuss the network architecture to process this data. First the 3D tensor *X* is passed through a sequence input layer. The next layer in the network is a bidirectional Long Short Term Memory (LSTM) (Yu et al., 2019) network. LSTM networks can capture long term dependencies in temporal data and has been successfully used for a number of time series classification problems. LSTM (similar to Recurrent Neural Networks, RNN; Sherstinsky, 2020) contains loops in its architecture which allows it to memorize previous states such that the network can effectively process temporal data. A typical LSTM layer consists of a set of recurrently connected blocks, known as memory blocks. Figure 5 shows the design of a typical LSTM unit or memory block. Each block contains one or more recurrently connected memory cell (*c*^*t*^) and three multiplicative units — the input (*i*^*t*^), output (*o*^*t*^), and forget gates (*f*^*t*^) which regulate the extent to which data is propagated through the LSTM unit. The operations inside an LSTM block can be formulated by the following:


$$\begin{array}{l} f_{t}=\rho \left(W_{f}x_{t}+U_{f}h_{t-1}+b_{f}\right)\\ i_{t}=\rho \left(W_{i}x_{t}+U_{i}h_{t-1}+b_{i}\right) \end{array}$$

(7) $$\begin{array}{l} o_{t}=\rho \left(W_{o}x_{t}+U_{o}h_{t-1}+b_{o}\right) \end{array}$$

$$\begin{array}{l} {ĉ}_{t}=\phi \left(W_{c}x_{t}+U_{c}h_{t-1}+b_{f}\right)\\ c_{t}=f_{t}⚬c_{t-1}+i_{t}⚬{ĉ}_{t}\\ h_{t}=o_{t}⚬\phi \left(c_{t}\right) \end{array}$$

Here *ρ* and ϕ are activation functions, ⚬ denote the element wise product operation, *x*_*t*_ is the input vector, *W* and *U* are weights and *h*_*t*_ is hidden state vector also known as output vector of the LSTM unit. Since the values of histogram for a certain lifetime component can be dependent of both past and future lifetime components, we use a Bidirectional LSTM(BLSTM), which includes both a forward and backward layer of LSTMs. Both the forward and backward layer outputs are calculated by using the standard LSTM update (Equation 7). Then BLSTM connects the two hidden layers to the same output layer. More details about BLSTMs can be found in Graves and Schmidhuber (2005) and Cui et al. (2020). The final layer of this subnetwork is a dropout layer, which randomly sets 50% of the input to 0.

### 2.3. Joint feature-LSTM network

The outputs of the morphological features subnetwork (RELU(BN((*W*_*c*_ϕ)), where *W*_*c*_ is the activation weights for the fully connected layer in that subnetwork) and the LSTM subnetwork (δ(*h*_*t*_) where *h*_*t*_ is the output of the final LSTM unit and the function δ(.) implements the dropout) is concatenated to produce a joint feature map. This is followed by a fully connected layer with 2 hidden unit (activation function *W*_*f*_) corresponding to each of the classes, a batch normalization layer(denoted by $\text{BN}(x)=\frac{x-E(x)}{\sqrt{(}Var(x))}$) and finally a Softmax layer with cross entropy loss($\sigma \left(y,ŷ\right)=-\frac{1}{n}\overset{n}{\underset{i=1}{\sum }}{ŷ}_{i}\log\left(y_{i}\right)$ where ŷ is the ground truth).


(8) $$\begin{array}{l} z=\text{concatenate}(\text{ReLU}\left(\text{BN}\left(\left(W_{c}\phi \right)\right)+\delta \left(h_{t}\right)\right) \end{array}$$

(9) $$\begin{array}{l} y=\mathrm{argmin}\left(\sigma \left(\text{BN}\left(W_{f}z\right),ŷ\right)\right) \end{array}$$

## 3. Materials

We conducted our experiments on two datasets of microglial cells containing both intensity images and corresponding lifetimes. The first data collected from mouse brain tissue samples consisting of 826 different cells, and the lifetime data collected across 256 time bins for each. We will refer to this as Dataset_1_. The second dataset is a collection of 506 samples from frontal cortex and hippocampus of mice, here the lifetime data is collected across 64 time bins. We refer to this dataset as Dataset_2_. For each dataset, we utilized the Becker&Hickl TCSPC (Time correlated single photon counting) software (Becker, 2021) to produce various parameters that includes the lifetime parameters and intensity images. We briefly elaborate on the acquisition details of the dataset in next section.

### 3.1. Tissue preparation and imaging

All animals were maintained in an AAALAC-accredited animal care facility with a 12-h light/dark cycle regime and had free access to food and water. All experiments were approved by the University of Wisconsin-Madison Institutional Animal Care and Use Committee (protocol V005173; exp. 5/16/2024).

#### 3.1.1. Dataset_1_ preparation and immunohistochemistry

One hundred micrometer thick coronal slices were generated from the fixed brains of 6–8 weeks old young adult male C57BL/6J and CX3CR1-GFP mice (Jackson Labs), to obtain the FLIM images. The mice were euthanized by isoflurane overdose and transcardially perfused with 30 ml of ice-cold PBS. This was followed by a second perfusion with an ice-cold solution of 4% PFA in PBS. After this, the brains were dissected and acutely post-fixed in 4% PFA prior to putting them into 30% sucrose in PBS overnight at 4°C until they sank. Brains were then stored in 15% sucrose/HBSS at −20°C prior to sectioning.

*Immunohistochemistry:* 100 μm thick coronal sections from the midbrain region of each brain was prepared using a Leica Vibratome. For immunohistochemical staining, two slices from each animal were used. These were washed with 0.3% TritonX-100 in PBS at room temperature, before incubating in blocking buffer (1% BSA, 0.3% 2 h also at room temperature). The slices were incubated with anti-Iba1 antibodies (1:1, 000; Wako Catalog No. 019-19741) in blocking buffer at 4°C overnight without light. This was followed by washing the slices at room temperature with 0.3% TritonX-100 in PBS. After this, the slices were incubated in the dark for 2 h with AlexaFlour594 anti-rabbit IgG antibodies (1:200) in blocking buffer, at room temperature. Slices were then washed with 0.3% TritonX-100 in PBS and mounted on 1mm slides using Cytoseal60 mounting medium. Finally the mounted sections were stored at room temperature and protected from light until imaging was done.

#### 3.1.2. Dataset_2_ preparation and immunohistochemistry

Dataset_2_, our second dataset, comprises CX3CR1-GFP mice (stock no 005582) ordered from Jackson laboratories (Bar Harbor, ME, USA). Mice were divided into two treatment groups and injected intraperitoneally with either 1 mg/kg lipopolysaccharide (LPS; Sigma-Aldrich) diluted in sterile Hanks Buffered Salt Solution (HBSS; Corning) or with vehicle (sterile 1xHBSS). There were 5 mice in each of the LPS treated, and vehicle treated groups. Animals were euthanized 16 h following vehicle or LPS injections and intracardially perfused with ice cold 1X Phosphate Buffered Saline (PBS) solution (30 mL per mouse). Mice were then perfused again with ice-cold 4% paraformaldehyde (PFA) in 1xPBS, pH 7.4. Brains were dissected intact, post-fixed for 24 h in a solution of 4% PFA in 1xPBS, then moved to 15% sucrose/1xHBSS (all performed at 4°C and protected from light).

Each brain was then cut into 100m thick coronal sections using a Leica vibratome. Slices were collected from regions of the brain containing frontal cortex and hippocampus for imaging (4 slices from each region). Slices were then mounted on 1-mm slides using Cytoseal604 (Richard-Allan Scientific, Kalamazoo, Michigan) mounting medium and 1.5 coverslips. The Cytoseal60 was allowed to cure for 24 h before sealing the edge of the slides with clear nail polish. Mounted sections were stored at room temperature, protected from light until imaging.

#### 3.1.3. Multiphoton lifetime imaging

The fluorescence lifetime (Lakowicz et al., 1992a) and multiphoton imaging (Denk et al., 1990) was performed on a custom multiphoton laser scanning system (built around an inverted Nikon Eclipse TE2000U) at the Laboratory for Optical and Computational Instrumentation (LOCI) in Madison, Wisconsin (Yan et al., 2006). A 20x air objective (Nikon Plan Apo VC, 0.75 NA) (Melville, NY, USA) was used for all imaging. The data was collected using an excitation wavelength of 740 nm for NAD(P)H, and the emission was filtered at 457/50 nm (Semrock, Rochester, NY) for the spectral peak. To identify the microglia, Iba1 (Ito et al., 1998) was used as the primary binding protein, whereas AlexaFluor594 was used as secondary protein. For generating the intensity images from FLIM, excitation was set at 810 nm, and a 615/20 (Semrock, Rochester, NY) bandpass emission filter was used for emission. For processing, we used Becker and Hickl time domain FLIM imaging software where decays curves are built with TCSPC (Time Correlated Single Photon Counting) electronics. FLIM images of 256 × 256 pixels (or 64 × 64 pixels) were collected with 120 s collection (for Dataset_1_) and 90 s (for Dataset_2_) using SPC-150 Photon Counting Electronics (Becker and Hickl GmbH, Berlin, Germany) and Hamamatsu H7422P-40 GaAsP photomultiplier tube (Hamamatsu Photonics, Bridgewater, NJ). To determine the Instrumentation Response Function (IRF), we used urea crystals with a 370/10 bandpass emission filter (Semrock, Rochester. NY) and was measured during each imaging session. In Dataset_1_, about 20 neighboring FOVs were chosen randomly, and the average lifetime value and free NADH ratio was calculated based on masking. For Dataset_2_, our second dataset, the entire region was imaged (Frontal Cortex or Hippocampus) and we selected the FOVs which that contained sufficiently bright microglia.

## 4. Experiments

We describe our evaluations in the following way: (1) First we obtain classification accuracy and other metrics using five-fold cross validation to quantitatively measure how well our model performs for the task of distinguishing activated microglia from resting in both datasets. (2) Secondly, we study the contribution of lifetime data in the joint model by comparing it against the performance of the network which uses only the shape features. (3) Third, we evaluate the performance of our model as a classifier by comparing it to other well-known classification schemes such as SVM, KNN and random forest. (4) Next, we evaluate the utility of each individual shape features that constitute our feature set and their combinations toward classification by comparing the classification results on a number of different subset of shape features. (5) We then study how the data is correlated across the two feature sets (shape and lifetime) by the observing their projection on canonical components found by CCA. (6) Finally, we evaluate the effect of training set size and model parameters toward the accuracy and efficacy of learning. Our models were implemented using Matlab's deep learning toolbox and Stochastic Gradient Descent (SGD) was employed to do the optimization. We discuss these issues next.

### 4.1. Evaluation of efficacy of joint morphology-lifetime based model

**Figure 6:**
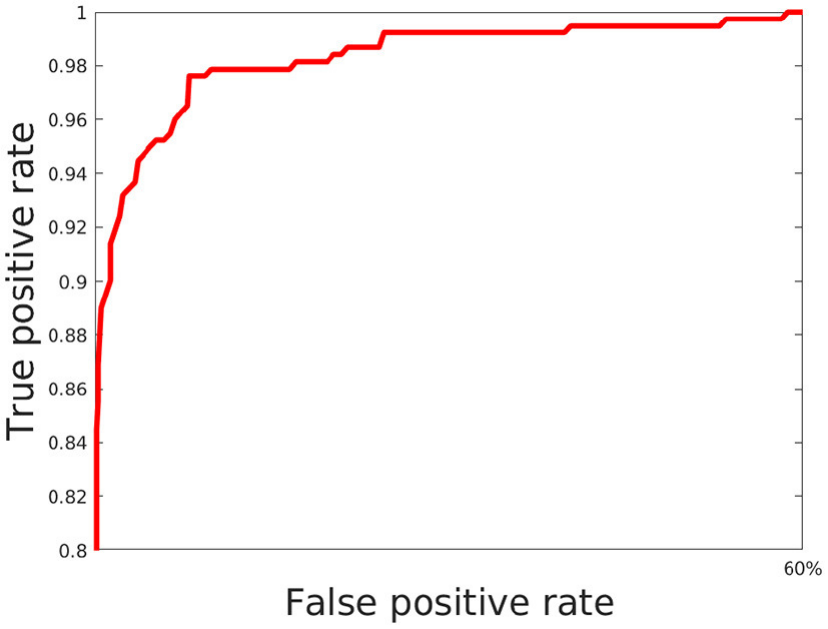
ROC for classification using our model.

Here, we train out model using 5 fold cross validation (this was performed by dividing the data into 5 equal parts and using 4 parts for training and one part for evaluation/validation of the model's performance. To choose the model parameters, such cross validation experiments were repeated for various settings of parameter values such as epoch and the setting with best accuracy was chosen). We use 6 different classification metrics to evaluate the quality of the classification with respect to ground truth. These include the Accuracy (Acc), Precision (prec), Recall (rec), Jaccard coefficients (JI), Area Under the Curve (AUC) as well as the ROC curve which plots true positive rate with respect to false positive rate (Figure 6). Results shown in Table 2 shows these metrics by averaging across all cross validation runs for the testing set. Analysis of these results show our model performs very well across all metrics and has both high accuracy as well as precision. This indicates that it is successfully able to distinguish activated microglia from resting for both datasets.

**Table 2:** Results showing performance of the joint lifetime+feature network on various metrics.

**Metric**	**Dataset_1_**	**Dataset_2_**
Accuracy	0.964	0.977
Precision	0.986	0.996
Recall	0.95	0.961
Jaccard index	0.938	0.958
AUC	0.966	0.981

### 4.2. Evaluation of utility of lifetime data

In this section, we study the impact of the lifetime data in the joint model. To do this, we use a separate network consisting of only of the subnetwork described in Section 2.1, which processes the shape feature data. We use similar 5-fold cross validation studies to obtain the accuracy results for both training and test and compare it to the results from the joint network in Table 3. The results show that while the shape based network performs well, accuracy improves by 2 − 3%, when lifetime data is included, which indicates it is a useful feature for this purpose.

**Table 3:** Results showing comparison of the shape+lifetime network with only feature based network.

	**Metric**	**Dataset_1_**	**Dataset_2_**
Shape + Lifetime	Train accuracy	0.971	0.981
	Test accuracy	0.972	0.978
Shape	Train accuracy	0.9472	0.977
	Test accuracy	0.9418	0.975

We also studied the utility of LSTM network in processing the lifetime data as a time series by comparing with the fitted life time data τ [given as τ = mean(*a*_1_τ_1_+*a*_2_τ_2_)] where the variables have the same meaning as in Equation 6. Since this converts lifetime into a feature, it can simply be concatenated to the existing feature set and then our subnetwork in Section 2.1 is used for learning. The results did not show any significant improvements compared to the features used without τ (Table 3, last two rows). This shows that using the individual values *a*_1_, *a*_2_, τ_1_ and τ_2_ for each lifetime component *t* is more useful for this purpose than a summary of the lifetime (using τ).

### 4.3. Comparison with other classifiers

Here, we discuss the performance of other classifiers on our datasets. We ran similar 5-fold cross validation studies by training SVM, KNN (with *K* = 3), an ensemble classifier and a feed-forward deep neural network (DNN) with two hidden layers each followed by a leaky ReLU and a softmax function as the output layer on both datasets. The SVM was trained with a Radial Basis Function (RBF) kernel whereas the ensemble classifier uses boosting to aggregate of 100 individual classification trees. Since there is no easy way to incorporate 3D time-series data similar to the format we are using in our network, in these classifiers, they were trained on shape features alone. We report on these results in Table 4. It shows that our model outperforms the other classifiers especially on Dataset_1_. Note that we should not compare the classifier scores across these two datasets, since we found that in general Dataset_2_ is easier to classify compared to Dataset_1_ as is evidenced by the higher accuracy and other metrics for some of the comparable methods used here.

**Table 4:** Comparison with other classifiers.

**Method**	**Dataset_1_**	**Dataset_2_**
Ours	0.972	0.978
SVM	0.8848	0.9644
KNN	0.9304	0.9605
Ensemble	0.9406	0.9723
DNN	0.9479	0.9684

### 4.4. Evaluation of individual shape features and their combinations

This experiment is aimed at validating the choice of shape feature set described earlier by comparing its performance with other several feature extraction approaches and their concatenation. To do this, we not only run the joint shape+lifetime model on General shape features (GenShape), Moment based features (Mom), Chord length histograms (ChordLen) and Elliptical Fourier features (ElliFou) individually, but also on various combinations of these features listed in Table 5. These features have been described earlier in Section 2.1. We see that Chord length histogram are the best individual feature whereas Moment based features perform the worst. The combinations of these feature gradually improve the performance and the best results are obtained when all four feature sets are concatenated to yield the feature set.

**Table 5:** Comparison of different feature subsets.

**Feature set**	**Dataset_1_**	**Dataset_2_**	**Feature set**	**Dataset_1_**	**Dataset_2_**
GenShape	0.86	0.845	Mom	0.545	0.562
ChordLen	0.96	0.95	ElliFou	0.918	0.8535
GenShape+Mom	0.957	0.958	GenShape+ChordLen	0.961	0.950
GenShape+ElliFou	0.963	0.9683	Mom+ChordLen	0.962	0.948
ChordLen+ElliFou	0.961	0.956	Mom+ElliFou	0.64	0.697
Mom+ChordLen+ElliFou	0.961	0.9624	GenShape+ChordLen+Mom	0.961	0.954
GenShape+ChordLen + ElliFou	0.958	0.960	All	0.964	0.977

### 4.5. Correlation of lifetime and feature data

**Figure 7:**
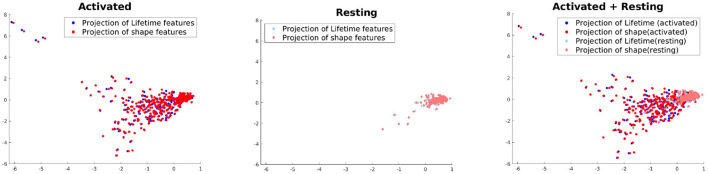
Projection using KCCA on the direction where correlation between the feature sets are maximized.

This experiment is not directly related to studying the performance of our model but aimed at understanding to what extent the shape and lifetime data used in this paper are correlated to each other across the two activation classes. For this purpose, we use Kernel Canonical Correlation Analysis (KCCA) (Hardoon et al., 2004) to find a common subspace where both of these feature sets are the most correlated. To apply KCCA, we need to construct kernels for both these feature sets. For the lifetime data, we compute the Wasserstein distance (a distance functions used for probability distributions) (Rüschendorf, 1985) of the histograms at each lifetime component for two samples *i* and *j*, which is then summed up across all lifetime components, to yield a distance *d*_*ij*_. The kernel is then constructed as $K_{ij}^{L}=exp\left(-td_{ij}\right)$, where one can tune if needed the bandwidth parameter *t* according to the learning task. For the features, kernel *K*^*F*^ is simply the gaussian kernel. We use the KCCA approach of Hardoon et al. (2004) to compute two directions of maximum correlation between these feature sets and project the data onto these subspaces. The results can be seen in Figure 7. It shows that the variance in the data is higher and the correlation between the feature sets are lower in case of activated vs. resting. This seems to intuitively explain the multiple morphological states which all can be evident in the activated state of microglia.

### 4.6. Evaluation of training set size network parameters

**Figure 8:**
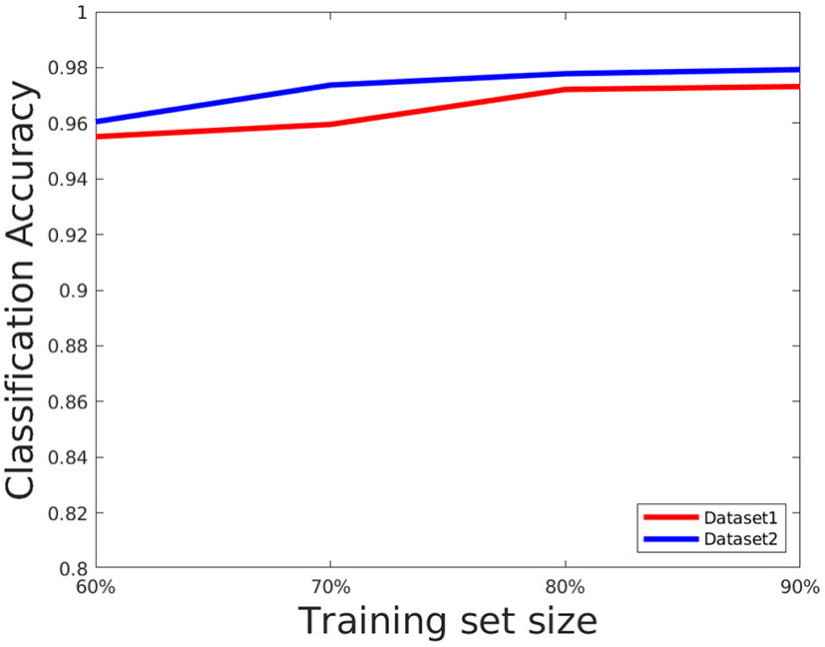
Plot showing how classification accuracy varies with training set size.

**Figure 9:**
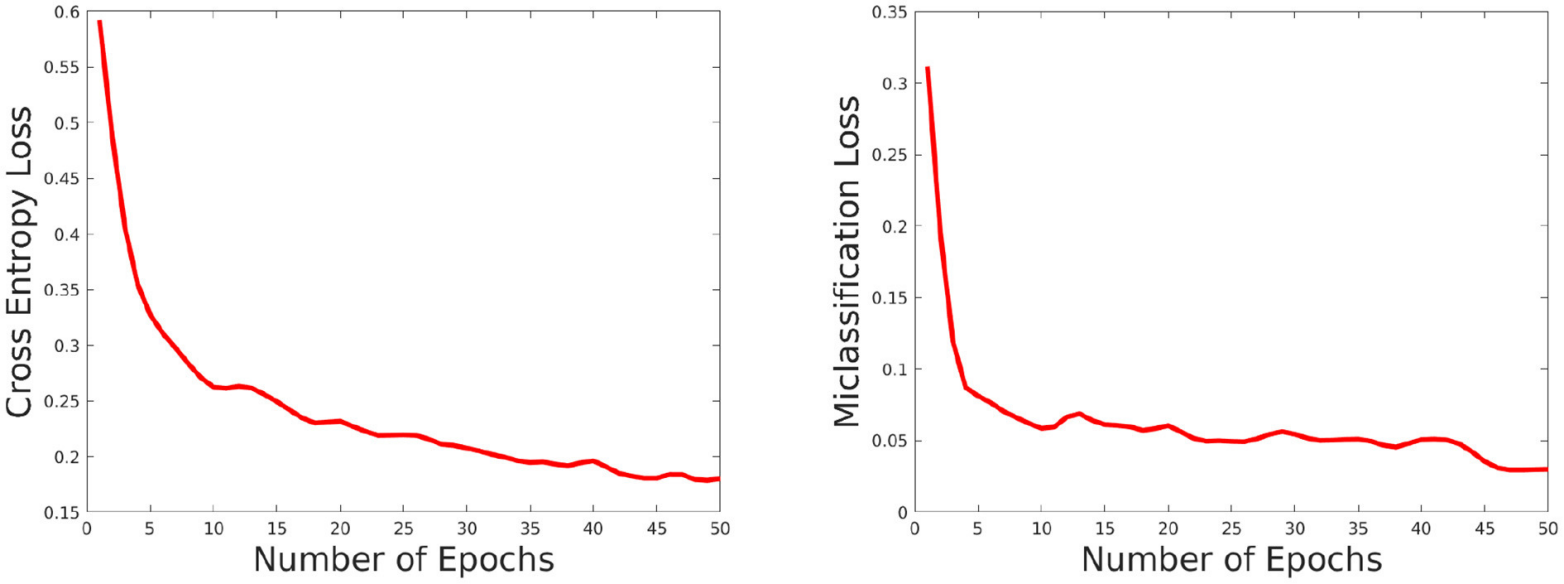
Plot showing how the cross entropy loss **(left)** and misclassification loss **(right)** varies with increasing epochs.

The network performance depends on various parameters such as learning rate, number of epochs used in training as well as size of the training set. We briefly discuss these issues here. We set the number of epochs to 50. Higher epochs lead to higher training and test accuracy but the running time in training is higher. The learning rate has been set to 0.0001, this value is also determined empirically as a trade-off of accuracy vs. running time. To evaluate the effect of the training set, we choose the training set size from the following set: {60, 70, 80, *and* 90%} of the overall data, and the rest of the data is considered for testing and evaluate the accuracy in each setting. The results are shown in Figure 8. As can be seen from this figure, the accuracy improves for a larger training size but change in magnitude is relatively small. This shows the model can give good performance even with a smaller training set. We also plotted the cross entropy loss and misclassification loss with respect to epochs in Figure 9. This shows that both losses show relatively small change in magnitude after about 20 epochs, so the number of epochs can be reduced without a substantial change in accuracy of the network.

## 5. Discussion

We have shown that our classification method of microglial activation status performs well by using morphology and lifetime metabolism data together. This study provides improved tools for researching microglia morphological changes and their corresponding responses that are associated with changes in the CNS microenvironment. There have been several recent developments investigating intensity-based data from tissue immunohistochemistry (Heindl et al., 2018) to analyze morphological shifts during the microglial inflammatory response. But to our knowledge, this is the first study that incorporates deep learning approaches to study microglia morphology in order to fully automate extraction of morphological information. This method can be further adapted to study microglia in various situations to characterize their activation state without requiring intensity-based morphological analyzes. In the future, we aim to study microglial activation in response to neurodegenerative disease, such as Alzheimer's disease, in which microglial inflammation is a hallmark. We postulate that more accurate classification of microglia functional states will lead to better prediction of early onset of neurodegenerative disease. Given how physiological inflammation affects immune cells differently; we believe our study could allow differentiation of microglia and macrophages or other CNS cell types. The novelty of combining microglial morphology with FLIM NADH measures adds an additional layer of tools with which to identify a microglia-specific cellular signature. This automated deep learning approach can also help distinguish the localized distribution of activated microglia to detect local inflammation.

In addition to the above mentioned future directions, below, we discuss some possible extensions to the methodological side of this approach as well as some challenges associated with this method.

*Challenges:* One of the greatest challenges which we inherit from other methods for automatically detecting cell morphology from fluorescence microscopy data is the uneven fluorescence detection across a field of view. Furthermore, because the shape feature detection module is dependent on the upstream segmentation of microglia, an error in the segmentation process can affect the quality of the morphological features obtained. Furthermore, the morphology analysis described above relies on segmentation of a two-dimensional projection that represents three dimensional volume, which is inherently a lossy transformation. In terms of computational issues, the time required for training increases with the size of the dataset, even though it was not an issue with the dataset used in this paper.

### 5.1. Possible extensions

1. To verify our classification method with regard to using shape features, we analyzed several morphological parameters obtained from 4 different types of shape features. Although this is more extensive than the types of features used in previous studies, in the context of microglia (Zanier et al., 2015; Fernández-Arjona et al., 2019), there are a lot more shape features that could also be used. Examples of such shape features include but are not limited to shapelets, wavelets, and Bag of contour fragments (BCF) (Wang et al., 2014). Since shape features are generated independently from our neural network, any of the above mentioned features can be easily integrated and tested with our model.
2. In this paper, we have focused on only two functional states of microglia (surveillant and reactive), however in terms of morphology alone, microglia are often classified into several different morphological classesc (Leyh et al., 2021). It would be interesting to extend our model to a multiclass classification framework to study how lifetime parameters correlate with different morphological classes.
3. As an additional step, we can study microglia from serial sections for 3D reconstruction of human brain tissue. For this, we would need to acquire volumetric features, but the rest of the algorithm can be applied without adjustments.

## 6. Conclusion

Using fluorescence lifetime imaging, here we propose an efficient approach to characterize microglial function/activation state, using a wide variety of shape features (far beyond what is commonly used in literature), together with metabolic characteristics of microglia. Our results show that this tandem analysis results in highly accurate predictions of microglial activation states, delivering state of the art results on over 1, 000 cell samples. Our proposed method for identifying microglial activation status using deep learning methods provides an unbiased, objective and computationally efficient approach that can serve as a useful tool for characterizing microglial functional and morphological transformations in the diseased CNS of animal models and humans alike. It also provides a baseline which can be extended by future studies that aim to apply deep learning algorithms toward identifying microglial subtypes and assess their accuracy.

## Data availability statement

The raw data supporting the conclusions of this article will be made available by the authors, without undue reservation.

## Ethics statement

The animal study was reviewed and approved by University of Wisconsin-Madison Institutional Animal Care and Use Committee.

## Author contributions

LM and MS contributed to the design and implementation of the study. JO is responsible for acquiring the dataset under the supervision of JW. KE is a senior author who contributed to developing of the main ideas and wrote sections of the paper. All authors contributed to manuscript revision, read, and approved the submitted version.
